# The Qualification Question

**Published:** 1890-05-17

**Authors:** 


### The Hospital

A Weekly Journal of

Science, Medicine, Nursing, and Philanthropy.

*For Hospitals, Asylums, Medical Practitioners, Students, Nurses, Families, Parishes, Congregations, and the Charitable Public.*

Vol. VIII.—No. 190.] [May 17, 1890.

### THE QUALIFICATION QUESTION.

A physician to a County Hospital writes to us complaining that the subject of qualifications for honorary hospital appointments is in a state of chaos; that few people seem to understand anything about it, and that nobody cares. With the first and second of these complaints we entirely agree; to the third we as decidedly demur. The problem of qualification for hospital appointments is one of the most fundamental in hospital administration. It raises first of all this question—What is the true and proper business of a hospital? And secondly—how may that business best be done? The business of the hospital two or three hundred years ago, and for many centuries before that time, was to furnish primarily an asylum for the sick, and then, so far as was possible, kind nursing and medical treatment. But is that the business of a hospital to-day? Decidedly not! Everybody is agreed now that the true use of a hospital is to provide the highest medical and surgical skill of the times, for the service of the most difficult cases, among the poorer classes of the people. To that primary and fundamental conception of the whole duty of a hospital there must in certain cases be added this—that the hospital is a place of instruction for those who are studying the science and art of medicine.

If it be admitted that our conception of the modern hospital is the right one, a very clear light is thrown upon the qualification question. Whatever a physician or surgeon to a hospital may or may not be as to academic status, he must imperatively be this—a man possessing adequate knowledge of the medical science of the times, and competent skill to put that science into practice. So obvious is our contention that no words need be wasted in its maintenance. There are other circumstances connected with the honorary medical staff which are of some importance. It is desirable, for example, that a hospital physician or surgeon shall be a man of sound and, if possible, of broad, literary culture. For if he be not, whatever may be his present knowledge and skill, he will not keep abreast of, much less advance, the medical science of his times. The result of that will be that his department of hospital work will fall behind, that his patients will be less well treated than at more advanced hospitals, and that in consequence of his influence the medical knowledge and skill of his whole neighbourhood will be more or less deteriorated.

A consideration which is often overlooked by hospital managers in making appointments, is the need for a certain amount of leisure in their candidate. London managers can perhaps hardly go wrong on this point; for, as a rule, their candidates are very liberally endowed in the matter of leisure. But in the country the case is different. There the temptation is to appoint a man on the ground of his extensive professional popularity. No doubt it often happens that the most popular doctor is also the best; though this is by no means universally the case. If a man already in large practice seeks a hospital appointment he should in every instance be required so to re-arrange his ordinary duties, as to be able to give ample time and full intellectual energy to his hospital work. Any physician or surgeon who is not prepared to do this should not covet a hospital appointment; he should either be content with what he has got, or make up his mind to forego some of the advantages of his present circumstances for the sake of the greater honour and higher prospects of the position he seeks. If, to the characteristics already indicated, it be added that the hospital physician or surgeon should also be a man of kind and dignified manners and of some experience of the world, our position on this part of the controversy will be defined with sufficient clearness.

It has not been very difficult to set forth the chief considerations which should weigh with hospital managers in the selection of officers to fill their honorary appointments. In human affairs it is often very easy to see what is required to be done, but very difficult to do it. The present is an example of such a case. Nevertheless, since the difficulty has so often to be faced in practice, we need not hesitate much about dealing with it in theory. On one point everybody is agreed, that nobody who is not a registered medical practitioner should be appointed honorary physician or surgeon to a hospital. Thus the range of choice is very decisively narrowed by universal consent. But, as everybody knows, there are practitioners and practitioners. There is the young man who, having spent three or four years behind a chemist's counter, suddenly develops an ambition to become a doctor. We say nothing against the ambition; it is entirely praiseworthy. But the young man has no money, and to become a doctor of ever so modest a kind requires a good deal of money, and a good deal of time as well. The young man must, therefore, limit his desires and ambitions in several important directions. He must seek a preliminary examination by way of entrance gate which requires the least possible amount of general education, and he must enter for a qualification which demands the narrowest range of study, the shortest period of pupilage, and the smallest measure of medical and surgical acquirements. On these lines the young chemist obtains a licence, such as it is, to practice medicine. Of course, he is not compelled to stop at this point. If his ambition, his capacity, and his patience be large enough, there is no reason why he should not in due time become President of the Royal College of Physicians. Usually, however, he remains throughout life an undistinguished Licentiate of one of the minor licensing bodies.

As contrasted with this young man, there is the public schoolboy who, at the age of eighteen, goes to Oxford or Cambridge, or, if he is not very rich, to London or Edinburgh. He begins with the intention of taking the M.D. degree, and if Oxford or Cambridge be his choice, the M.A. as well. He, therefore, enters with the best preliminary education, upon the largest range of study, for the longest period of pupilage, and for the most numerous and difficult qualifying examinations. Between a doctor of medicine thus educated and examined and an apothecarial licentiate there is every conceivable variety of qualification. From nearly thirty thousand men thus variously qualified every hospital has, or ought to have, unrestricted range of choice. But though that is so, it is very plain that hospital managers who are wise will look mostly in one direction for their honorary officers. They will naturally desire that all of them, surgeons as well as physicians, shall have university qualifications. There is no doubt that, including the three elements of general culture, scientific attainments, and professional knowledge, the university man stands at the head of all his rivals. We make no pretence of distinguishing between the value of different university degrees. That is a childish pastime which may safely be left to undergraduates. But though we do not insist that every hospital physician shall be a university man, we do insist that the only justification for appointing a man of inferior academic status must be that of proved professional superiority.

Practically, then, we have given our judgment in favour of university degrees for all honorary medical officers of hospitals, whether physicians or surgeons. Ought any other qualification to be considered as on an all-round level with these? No! But there are some that are hardly inferior to them. A member or fellow of a Royal College of Physicians, and a member or fellow of a Royal College of Surgeons, more particularly a fellow, may be regarded as next in professional rank to the university man. In any actual case of proved personal and professional superiority such a member or fellow of a Royal College should be appointed before the university graduate without hesitation. It is a common practice with hospitals to demand of their candidates both university and Royal College qualifications. This is quite unnecessary in practice and logically absurd. One high-class qualification is as good as a dozen. The man who has had the industry and capacity to take the one could take a hundred if his time and his pocket permitted. Having acquired the necessary discipline and knowledge implied in the obtaining of his single first-rate qualification, he can spend his time to much better purpose than in running about from one examination board to another, in order to gratify an unmanly craving for useless professional distinctions. The soldier whose heart is set upon medals is generally a poor, vain creature. Space fails for the discussion of the question more at length, though we fully recognise its importance, and may return to it as occasion serves.

